# Self-reported health and health care use in an ageing population in the Agincourt sub-district of rural South Africa

**DOI:** 10.3402/gha.v6i0.19305

**Published:** 2013-01-24

**Authors:** Francesc Xavier Gómez-Olivé, Margaret Thorogood, Benjamin Clark, Kathleen Kahn, Stephen Tollman

**Affiliations:** 1 MRC/Wits Rural Public Health and Health Transitions Research Unit (Agincourt), School of Public Health, Faculty of Health Sciences, University of the Witwatersrand, Johannesburg, South Africa; 2 INDEPTH Network, Accra, Ghana; 3 Warwick Medical School, University of Warwick, Coventry, UK; 4 Centre for Population Studies, London School of Hygiene and Tropical Medicine, London, UK; 5 Centre for Global Health Research, Epidemiology and Global Health, Umeå University, Umeå, Sweden

**Keywords:** health care use, older population, self-reported health, non-communicable disease, WHODAS, WHOQOL, rural, South Africa

## Abstract

**Background:**

South Africa is experiencing a demographic and epidemiological transition with an increase in population aged 50 years and older and rising prevalence of non-communicable diseases. This, coupled with high HIV and tuberculosis prevalence, puts an already weak health service under greater strain.

**Objective:**

To measure self-reported chronic health conditions and chronic disease risk factors, including smoking and alcohol use, and to establish their association with health care use in a rural South African population aged 50 years or older.

**Methods:**

The Study on Global Ageing and Adult Health (SAGE), in collaboration with the INDEPTH Network and the World Health Organization, was implemented in the Agincourt sub-district in rural northeast South Africa where there is a long-standing health and socio-demographic surveillance system. Household-based interviews were conducted in a random sample of people aged 50 years and older. The interview included questions on self-reported health and health care use, and some physical measurements, including blood pressure and anthropometry.

**Results:**

Four hundred and twenty-five individuals aged 50 years or older participated in the study. Musculoskeletal pain was the most prevalent self-reported condition (41.7%; 95% Confidence Interval [CI] 37.0–46.6) followed by hypertension (31.2%; 95% CI 26.8–35.9) and diabetes (6.1%; 95% CI 4.1–8.9). All self-reported conditions were significantly associated with low self-reported functionality and quality of life, 57% of participants had hypertension, including 44% of those who reported normal blood pressure. A large waist circumference and current alcohol consumption were associated with high risk of hypertension in men, whereas in women, old age, high waist–hip ratio, and less than 6 years of formal education were associated with high risk of hypertension. Only 45% of all participants reported accessing health care in the last 12 months. Those who reported higher use of the health facilities also reported lower levels of functioning and quality of life.

**Conclusion:**

Self-reported chronic health conditions, especially hypertension, had a high prevalence in this population and were strongly associated with higher levels of health care use. The primary health care system in South Africa will need to provide care for people with non-communicable diseases.

Hypertension in developing countries has been an area of concern for many years (1) and several reviews have called attention to this public health problem (2, 3). In 2000 a study in South Africa showed that high blood pressure was responsible for 9% of all deaths and 2.4% of all Disability Adjusted Life Years (4). A recent research article has described the impact on health care demand of the increase in non-communicable diseases in South Africa (5). The United Nations General Assembly addressed this global problem, proposing new global interventions to attend to the increasing world-wide burden of non-communicable diseases, promote prevention and strengthen health services (6). In September 2010, the South African Summit on the Prevention and Control of Non-communicable diseases acknowledged the impact that the coming epidemic of non-communicable disease will have in South Africa (7).

African populations are ageing and undergoing demographic and health transition (8). The resulting South African demographic and health changes have led to an increasing older population with some 15% of the population aged 50 years and older and 7.7% aged 60 years and older in 2011 (9). Older age groups are known to experience a high prevalence of non-communicable diseases, and so an increasing burden of these conditions can be expected (5, 10). Although a study in 1998 found high levels of hypertension in South Africa, particularly in urban areas (11), there is no routine measurement of the prevalence of non-communicable diseases in South Africa that could help measure this change (12). The annual District Health Barometer included diabetes and hypertension for the first time in its 2010/2011 annual report, recognising both the lack of morbidity data and the importance of non-communicable diseases in South Africa (13).

The primary health care system in South Africa is inadequately prepared to meet new demands for care of non-communicable diseases in addition to up-scaling antiretroviral therapy for HIV and AIDS patients (14). In recognition of these challenges, the South African Ministry of Health has started to re-engineer the primary health care system including addressing issues of integrated chronic disease care (8), accessibility (15, 16), and acceptability. However, more information on the health care needs of older people and their current use of health care systems is needed.

The data for this study were collected before the recent health care reforms. This cross-sectional study aims to describe self-reported non-communicable diseases, and self-reported smoking and alcohol use, measures of blood pressure and body size, together with health care use of an older population living in a rural South African area.

## Methods

The Study on Global Ageing and Adult Health (SAGE) is a collaboration between the INDEPTH Network and the World Health Organization (Department of Health Statistics and Informatics). The study was run at national level in six countries (India, China, Russia, Ghana, South Africa, and Mexico) and at three INDEPTH Health and Demographic Surveillance System (HDSS) sites in India (Vadu HDSS), Ghana (Navrongo HDSS), and South Africa (Agincourt HDSS). This article presents an analysis of SAGE data collected in the Agincourt HDSS site.

### Setting

The study was based in the Agincourt sub-district of Mpumalanga Province, South Africa. Since 1992, the MRC/Wits Rural Public Health and Health Transitions Research Unit (Agincourt) has collected data on the sub-district population, with vital events (pregnancy outcome, deaths, and migrations) updated yearly by trained local fieldworkers (17). Additional data (labour participation, household assets, education status, and union status) are collected at different time intervals to complement demographic data and give contextual information. The total population under surveillance in 2006 was approximately 72,801 people distributed in 21 villages and 11,734 households, of which 8,224 (11.3%) were aged 50 years or older.

Since 1992, there has been substantial socio-economic development in the area. However, the unemployment rate remains high with 60% labour migration among men aged 35–54 years and an increasing proportion of labour migrants among young men and women (17). The gender distribution of people permanently living in the area is affected by this migration, resulting in a higher proportion of resident females.

The public health system in the sub-district consists of six clinics and one health centre. Hospital services are covered by three hospitals situated between 25 and 45 km from the study site (18). At the time that the data described in this article were collected, clinics did not have specific services for chronic diseases and the registration and follow up of patients was erratic, with no system for identification and recall of patients who did not attend monthly routine appointments to get medication. Although primary health care services are free, the cost of transport may reduce the attendance at health facilities. Private doctors practice in the area and there is an active network of traditional healers (15, 19). At the time of the study, voluntary counselling and testing (VCT) services for HIV/AIDS were available in local clinics but antiretroviral treatment was only available in the district hospitals, and only a small proportion of the sub-district population was accessing this service.

The SAGE study was embedded within the Agincourt HDSS routine operations and the sample was selected from the population database. Information collected in this study could therefore be linked with existing socio-demographic variables.

### Sample

A random sample of 575 people was drawn from the 2005 update of the population database. The size of the sample was determined by the requirements of the larger WHO-INDEPTH international study. Inclusion criteria were residence in the study site for 12 months prior to the 2005 update round and being 50 years old or more at the beginning of the study on 1 May 2006. Men and women were selected separately reflecting the unequal proportions of men and women in the population aged 50 years and over.

### Quality control and training

A week of training for the principal investigators and researchers involved in multi-country SAGE studies was organised by WHO a few months before implementation of the study. Training focused on using and understanding the questionnaire manuals, standardising data collection, anthropometric measurements, and performance tests. Thereafter, the field supervisors, quality checkers, and fieldworkers on-site in Agincourt received 2 weeks training.

A quality control system was established with three control points in the field and validation checks in the data entry process.

### Data collection

The SAGE questionnaire (20) was translated to the local language (Xitsonga) and translated back to English. Additional demographic data, including gender, age, marital status, nationality, and level of education were extracted from the Agincourt HDSS 2005 database.

### Data entry, data cleaning, and analysis

Data were double-entered using CSPro 3.1 (U.S. Census Bureau) using an application provided by WHO which included validation checks. The data cleaning process included checking for completeness, identification and correction of outliers, and ensuring individual consistency on all ID codes. Data were then extracted to Stata11 (Stata Corp, College Station, Texas, USA) for analysis.

### Variables

We calculated age as of May 2006 and assigned participants to 5-year age groups. Years of formal education were obtained from the 2005 Agincourt HDSS database and categorised according to WHO levels of education: 6 years or more; less than 6 years, and no formal education. Marital status was categorised into two groups:

1. Current partnership: currently married or in an informal union; and
2. Single: those who had never married or those who were separated, divorced, or widowed.

We used data on employment status from the 2004 HDSS database since these were the most recent data available.

Socio-economic status (SES) was derived from the 2005 household asset survey. An SES indicator was constructed giving equal weight to each asset and rescaling it so that values of a given asset variable fall within the range [0, 1]. The assets were then categorised into five groups: ‘quality of housing’, ‘water and sanitation’, ‘power supply’, ‘modern assets’, and ‘livestock assets’. These rescaled asset values were added up for each household and asset group and rescaled again to give a specific value in the range [0, 1]. Finally, these five group-specific scaled values were totalled for each household, providing an overall asset score with a value in the range of [0, 5] (21). Household asset scores were grouped into quintiles for the entire Agincourt sub-district population. Participants in this study are not equally distributed across the five quintiles because they are a sub-sample of the whole population (50 years and older).

Before 1993, the Agincourt sub-district received a high number of refugees from Mozambique fleeing the civil war. Mozambican residents are separately identified in the HDSS database and differ from the host South African population in measures, such as education, household assets, and child mortality (22). The variable nationality of origin (South African/Mozambican) records the different origins of the study population.

The physical and social functioning of each participant was measured using the WHODAS II scale (World Health Organization Disability Assessment Schedule II) which assesses daily functioning. Ten questions on the difficulty experienced by the respondent when performing certain activities in the last 30 days were used to create the WHODAS II scale. The score ranges between 0 and 100 with a high score indicating severely impaired physical function. The Word Health Organization Quality of Life (WHOQOL) scale of 8 to 40 (where 8 is the best quality of life) was used. It includes questions on self-rated general health and on satisfaction with life. Both scales are explained in more detail elsewhere (23–26). We used two single questions to measure self-reported health and function. Participants were asked ‘In general, how would you rate your health today?’ with the options of very good, good, moderate, bad, and very bad; and ‘Overall in the last 30 days, how much difficulty did you have with work or household activities?’ with options being none, mild, moderate, severe, and extreme/cannot do.

Two life-style behaviours, cigarette smoking and consuming alcohol, are known to be associated to the occurrence of several chronic diseases. We defined ‘currently smoking’ as presently using any tobacco product even if not daily. We defined ‘currently drinking’ as having drunk any alcoholic beverage in the last 30 days.

Weight and height were measured and used to calculate the body mass index (BMI) using the formula: Weight in kilograms/height in metres^2^. A normal scale was used and weight was recorded to the nearest 0.1 kg. Height was measured using a stadiometer and measures were recorded to the nearest 0.1 cm.

Waist/hip ratio was calculated by dividing waist circumference in centimetres by hip circumference in centimetres. Waist circumference was measured at the navel level and the hip at the hip joint level. Both measures were taken using an inelastic tape and recorded to the nearest 0.1 cm.

Blood pressure was calculated by the average of the second and third blood pressure measurements taken. Hypertension was classified as follows:

1. Normal (SBP < 120 mmHg and DBP < 80 mmHg) and Pre-hypertension (SBP 120–139 mmHg or DBP 80–89 mmHg);
2. Stage 1 Hypertension (SBP 140–159 mmHg or DBP 90–99 mmHg); and
3. Stage 2 Hypertension (SBP ≥ 160 mmHg or DBP ≥ 100 mmHg) (27).

When blood pressure is presented as a binary variable, Stage 1 and Stage 2 Hypertension are combined as Hypertension.

Participants were asked whether they had ever been told that they had any of the following health conditions: arthritis, back pain, pain or swelling around joints, stroke, angina, diabetes, respiratory diseases, depression, and hypertension. We created a composite variable termed ‘musculoskeletal pain’ to include self-reported:

1. arthritis;
2. pain, aching, stiffness or swelling in or around the joints not related to an injury and that lasted for more than a month; and
3. back pain during the last 30 days.

We asked participants when was the last time they needed health care; if they had used care in the last 12 months; what type of health facility they used; and the reason for the visit.

### Statistical analysis

We explored factors associated with the presence of hypertension by constructing logistic regression models separately for men and women.

We entered age group *a-priori* into the multivariate regression model and then tested for the effect of all potential risk factors: BMI, waist circumference, waist–hip ratio, tobacco use, alcohol use, union status, education level, household asset status, nationality, physical function, and quality of life. Risk factors were sequentially introduced into the model and retained in the model if their inclusion had a significant effect at *p*<0.05.

## Results

We obtained completed questionnaires from 425 (73.9%) participants. We were unable to contact 118 (20.5%) participants who had out-migrated or were not available on the three occasions we visited them. In addition, 22 (3.8%) individuals had died and 10 (1.7%) declined to participate.

Women were significantly more likely to be single (*p*<0.001) (Table 1), report greater levels of disability (*p*=0.001) and lower quality of life (*p*=0.029) than men. There was weaker evidence that women rated their health as poorer (*p*=0.069) and reported more difficulty in performing daily activities (*p*=0.07) (Table 1). Men reported higher levels of smoking (p < 0.001) and higher levels of alcohol intake (*p*<0.001) (Data not shown).

**Table 1:** Socio-demographic measures, quality of life, function and life style by gender in the Agincourt sub-district in 2006

	Male	Female	Total	χ^2^ *p*-value (degree of freedom)
Five year age group				
50–54	20 (14.7)	64 (22.2)	84 (19.8)	0.525
55–59	27 (19.9)	46 (16.0)	73 (17.2)	(6 d.f.)
60–64	20 (14.7)	39 (13.5)	59 (13.9)	
65–69	26 (19.1)	42 (14.6)	68 (16.0)	
70–74	13 (9.6)	31 (10.8)	44 (10.4)	
75–79	16 (11.8)	39 (13.5)	55 (13.0)	
80 plus	14 (10.3)	27 (9.4)	41 (9.7)	
Union status*				
Currently married	98 (72.1)	102 (35.3)	200 (47.1)	<0.001
Single	38 (27.9)	187 (64.7)	225 (52.9)	(1 d.f.)
Years of education**				
No education	79 (59.9)	193 (68.7)	272 (65.9)	0.207
Less than 6 years	33 (25.0)	56 (19.9)	89 (21.6)	(2 d.f.)
Six years or more	20 (15.2)	32 (11.4)	52 (12.6)	
Employment status&				
Employed	35 (29.7)	34 (18.0)	69 (22.5)	0.058
Unemployed	7 (5.9)	13 (6.9)	20 (6.5)	(2 d.f.)
Retired/sick/family supported	76 (64.4)	142 (75.1)	218 (71.0)	
Nationality of origin				
South African	109 (80.2)	207 (71.6)	316 (74.4)	0.061
Mozambican	27 (19.9)	82 (28.4)	109 (25.7)	(1 d.f.)
Household asset score (2005)#				
Lowest	17 (12.6)	44 (15.3)	61 (14.5)	0.385
Middle low	23 (17.0)	49 (17.1)	72 (17.1)	(4 d.f.)
Middle	24 (17.8)	69 (24.0)	93 (22.0)	
Middle high	33 (24.4)	64 (22.3)	97 (23.0)	
Highest	38 (28.2)	61 (21.3)	99 (23.5)	
WHO Disability Adjusted Score (WHODAS II)†				
Very good	48 (35.3)	59 (20.4)	107 (25.2)	0.001
Good	28 (20.6)	40 (13.8)	68 (16)	(4 d.f.)
Mild	22 (16.2)	58 (20.1)	80 (18.8)	
Bad	19 (14)	71 (24.6)	90 (21.2)	
Very bad	19 (14)	61 (21.1)	80 (18.8)	
WHO Quality of life (WHOQOL)††				
Very good	47 (34.6)	65 (22.5)	112 (26.4)	0.029
Good	28 (20.6)	53 (18.3)	81 (19.1)	(4 d.f.)
Mild	23 (16.9)	48 (16.6)	71 (16.7)	
Bad	19 (14)	67 (23.2)	86 (20.2)	
Very bad	19 (14)	56 (19.4)	75 (17.7)	
Rate health today¥				Fisher exact test
Very good	13 (9.6)	17 (5.9)	30 (7.1)	0.069
Good	63 (46.3)	114 (39.5)	177 (41.7)	
Moderate	50 (36.8)	113 (39.1)	163 (38.4)	
Bad	10 (7.4)	44 (15.2)	54 (12.7)	
Very bad	0 (0)	1 (0.4)	1 (0.2)	
Difficulty work/household activities¥¥				Fisher exact test
None	50 (37)	71 (24.6)	121 (28.5)	0.07
Mild	36 (26.7)	80 (27.7)	116 (27.4)	
Moderate	26 (19.3)	82 (28.4)	108 (25.5)	
Severe	18 (13.3)	47 (16.3)	65 (15.3)	
Extreme	5 (3.7)	9 (3.1)	14 (3.3)	

* Union status: currently married includes official and traditional unions. Single includes never married, divorced, and separated

** Years of education: completed years of schooling

& Employment status: employment situation for those who worked in the past. Employed are currently working. Unemployed are those not currently working but looking for a job

# Household asset score: household weighted measured in quintiles used as a proxy to calculate socio-economic status. Data are from 2005

† WHODAS II (World Health Organization Disability Assessment Schedule II) is a self-rated measure of functionality presented in quintiles

†† WHOQOL (World Health Organization quality of life) is self-rated general health and questions on life satisfaction presented in quintiles

¥ Rate health today: is a self reported measure of health answering the question *‘*in general how would you rate your health today?*’*

¥¥ Rate difficulty work/household activities: is a self reported measure of functionality answering the question *‘*overall in the last 30 days, how much difficulty did you have with work or household activities?*’*

Overall, 42% of participants reported musculoskeletal pain, 31% hypertension, and 6% diabetes. Men reported respiratory diseases significantly more than women (*p*=0.039) and women reported hypertension significantly more than men (*p*=0.001) (Table 2).

**Table 2:** Prevalence of self reported health conditions by gender

	Male	Female	Total	

	Prevalence (N)	Prevalence (N)	Prevalence (N)	*p*
Musculoskeletal pain	38.52 (52)	43.21 (124)	41.71 (176)	0.362**(1 d.f.)
Stroke	1.48 (2)	1.74 (5)	1.65 (7)	0.604*
Angina	5.19 (7)	3.47 (10)	4.02 (17)	0.403**(1 d.f.)
Diabetes	6.67 (9)	5.9 (17)	6.15 (26)	0.76** (1 d.f.)
Respiratory disease	8.15 (11)	3.47 (10)	4.96 (21)	0.039** (1 d.f.)
Depression	6.67 (9)	4.86 (14)	5.44 (23)	0.445** (1 d.f.)
Hypertension	20.74 (28)	36.11 (104)	31.21 (132)	0.001** (1 d.f.)

* Fisher's exact test

** Chi square test.

We compared self-reported hypertension with measured blood pressure. Of the 255 participants who did not report that they had hypertension, high blood pressure levels compatible with hypertension were present in 112 (43.9%) (*p*<0.001). Half of the 20 participants who reported hypertension but were normotensive when their blood pressure was measured were on treatment for hypertension. The positive predictive value of a self-reported diagnosis of hypertension was 91.9%, but the negative predictive value of a self-reported normal blood pressure was only 43.9%. Awareness of diagnosis of hypertension varied with gender. Of the 262 women who had measured hypertension, 81 (30.9%) reported that they had hypertension, while of the 116 men with measured hypertension only 22 (19%) reported that they had hypertension (*p*=0.025).

We found that in all cases, individuals reporting one of the three most commonly reported diagnoses (musculoskeletal pain, hypertension, and diabetes) were significantly more likely to report bad or very bad functional ability (measured by WHODAS) and quality of life (measured by WHOQOL) (data not shown).

Table 3 shows the measures of body size and blood pressure in men and women. Women had a significantly higher mean BMI (*p*<0.001) and waist circumference (*p*<0.001) but the difference in waist/hip ratio was not significant (*p*=0.081). Despite the difference in body size, men and women had similar levels of blood pressure and similar proportions of participants with measured high blood pressure (Table 3). Mean systolic blood pressure (SBP) was 134.2 in both genders with 95% CI 129.8–138.5 for men and 95% CI 131.4–137.0 for women. When blood pressure levels were analysed by age group and sex, there was little evidence of a trend of increasing blood pressure with age in men, but a significant increase with age in women (data not shown).

**Table 3:** Anthropometric measurements and clinical measures by gender in the Agincourt sub-district in 2006

	Male	Female	

	N (%)	N (%)	*p*
Categories of BMI			
Underweight <18.5	11 (8.87)	4 (1.49)	<0.001*
Normal weight (18.5–24)	54 (43.55)	69 (25.65)	
Overweight (25–29.9)	37 (29.84)	82 (30.48)	
Obese class I (30–34.9)	19 (15.32)	67 (24.91)	
Obese class II (35–39.9)	1 (0.81)	24 (8.92)	
Obese class III (≥40)	2 (1.61)	23 (8.55)	
Waist/hip ratio category (WHO)			
Normal	36 (26.47)	55 (19.03)	0.081**
Substantially increased	100 (73.53)	234 (80.97)	1 d.f.
Waist circumference (WHO)			
Normal	73 (53.68)	28 (9.69)	<0.001**
Increased	25 (18.38)	47 (16.26)	1 d.f.
Substantially increased	38 (27.94)	214 (74.05)	
Blood pressure JNC7#			
Normal BP, no treatment	50 (43.1)	114 (43.35)	0.117**
Normal BP on treatment	7 (6.03)	23 (8.75)	5 d.f.
Stage 1 hypertension	22 (18.97)	46 (17.49)	
Stage 1 hypertension on treatment	0 (0)	12 (4.56)	
Stage 2 hypertension	32 (27.59)	52 (19.77)	
Stage 2 hypertension on treatment	5 (4.31)	16 (6.08)	

* Fisher exact test

** Chi square test

# JNC7: classification was done using US Department of Health and Human Services. Seventh report of the Joint National Committee on prevention, detection, evaluation and treatment of high blood pressure. 2004.

Reported use of health care facilities (See Table 4) was associated with a number of factors although, contrary to our expectations, we did not find any significant association between old age and use of health facilities. Women (*p*=0.041), those who were single (*p*=0.012), and those who were South African rather than Mozambican (*p*=0.005) were all more likely to use primary health care facilities. A higher level of education was also associated with greater use of health care facilities but this difference was not significant (*p*=0.066). Individuals reporting that they had a non-communicable disease were more likely to have used a health facility, as were those reporting a greater level of disability (*p*=0.001) or a lower quality of life (*p*=0.001).

**Table 4:** Health facility use (outpatient) by socio-demographic variables in the Agincourt sub-district in 2006

	No use	Only once	Two or more	

	N (%)	N (%)	N (%)	*p*
Gender				
Male	81 (60)	36 (26.7)	18 (13.3)	0.041β
Female	150 (52.0)	69 (24.0)	69 (24.0)	2 d.f.
Age groups				
50–54	44 (52.4)	22 (26.2)	18 (21.4)	0.962β
55–59	44 (60.3)	18 (24.7)	11 (15.1)	12 d.f.
60–64	28 (48.3)	15 (25.9)	15 (25.9)	
65–69	38 (56.7)	14 (20.9)	15 (22.4)	
70–74	26 (59.1)	11 (25.0)	7 (15.9)	
75–79	29 (52.7)	13 (23.6)	13 (23.6)	
80 plus	21 (51.2)	12 (29.3)	8 (19.5)	
PHC clinic in village@				
No PHC	100 (53.8)	49 (26.3)	37 (19.9)	0.817β
PHC present	130 (55.1)	56 (23.7)	50 (21.2)	2 d.f.
Union status*				
Currently married	123 (61.8)	38 (19.1)	38 (19.1)	0.012β
Single	108 (48.2)	67 (29.9)	49 (21.9)	2 d.f.
Years of education**				
No education	158 (58.5)	61 (22.6)	51 (18.9)	0.066β
Less than 6 years	45 (50.6)	23 (25.8)	21 (23.6)	4 d.f.
Six years or more	20 (38.5)	20 (38.5)	12 (23.1)	
Employment status&				
Employed	40 (58.0)	14 (20.3)	15 (21.7)	0.199α
Unemployed	12 (60.0)	7 (35.0)	1 (5.0)	
Retired/sick/family	106 (48.9)	60 (27.7)	51 (23.5)	
Nationality of origin				
South African	161 (51.0)	79 (25.0)	76 (24.1)	0.005β
Mozambican	70 (65.4)	26 (24.3)	11 (10.3)	2 d.f.
Household asset score (2005)#				
Lowest	38 (63.3)	16 (26.7)	6 (10.0)	0.28β
Middle low	37 (51.4)	22 (30.6)	13 (18.1)	8 d.f.
Middle	56 (60.9)	16 (17.4)	20 (21.7)	
Middle high	49 (50.5)	25 (25.8)	23 (23.7)	
Highest	50 (50.5)	26 (26.3)	23 (23.2)	
WHODAS†				
Very good/good	106 (60.9)	51 (29.3)	17 (9.8)	<0.001β
Middle	43 (53.8)	16 (20.0)	21 (26.3)	4 d.f.
Bad/very bad	82 (48.5)	38 (22.5)	49 (29.0)	
WHOQOL††				
Very good/good	122 (63.5)	51 (26.6)	19 (9.9)	<0.001β
Middle	39 (54.9)	14 (19.7)	18 (25.4)	4 d.f.
Bad/very bad	70 (43.8)	40 (25.0)	50 (31.3)	
Self-reported health conditions¥				
Stroke	3 (42.9)	1 (14.3)	3 (42.9)	0.008α
Musculoskeletal pain	66 (37.5)	49 (27.8)	61 (34.7)	<0.001β
Angina	9 (52.9)	4 (23.5)	4 (23.5)	0.02α
Diabetes	10 (38.5)	5 (19.2)	11 (42.3)	<0.001α
Respiratory disease	8 (38.1)	5 (23.8)	8 (38.1)	<0.001α
Depression	9 (39.1)	9 (39.1)	5 (21.7)	0.001α
Hypertension	43 (32.6)	40 (30.3)	49 (37.1)	<0.001β

α Fisher's exact test

β Chi square test

@ PHC in village: comparison of villages with a primary health care facility and those without

* Union status: currently married includes official and traditional unions. Single includes never married, divorced, and separated

** Years of education: completed years of schooling

& Employment status: employment situation for those who worked in the past. Employed are currently working. Unemployed are those not currently working but looking for a job

# Household asset score: household weighted measured in quintiles used as a proxy to calculate socio-economic status. Data from 2005

† WHODAS II (World Health Organization Disability Assessment Schedule II) is a self-rated measure of functionality presented in quintiles

†† WHOQOL (World Health Organization quality of life) is self-rated general health and questions on life satisfaction presented in quintiles

¥ Self reported health conditions: health conditions reported by respondent as being diagnosed in the 12 months previous to the interview. Analysis is on health facility use comparing those who reported a health condition with those not reporting any health condition.

Those participants who reported being told they had a health condition were asked if they had used any medication for the condition in the last 12 months. Of those reporting having diabetes, 98.3% said they had used medication at some time in the last 12 months, with slightly lower figures for other conditions (80.9% of those reporting lung disease; 87.0% of those reporting depression; 80.3% of those reporting hypertension). When asked about their use of health care, 86 participants (20.3%) reported that they had never needed care, and a further 96 (22.7%) reported that they had not needed care within the last 12 months. Twenty eight (32.5%) of those who reported never needing care nevertheless reported that they had a chronic health condition, including musculoskeletal pain (*n*=16), diabetes (*n*=6), and hypertension (*n*=6).

Information was available for 222 of the 241 people who reporting needing health care in the previous 12 months. Of the 222, 191 (86.0%) had received care. Of these 191, 19% received care in private facilities of various kinds, 22% in public hospital outpatient departments, and 59% in public clinics.

**Table 5:** Risk factors for high blood pressure in people aged 50 years or older in a rural South African setting (Agincourt sub-district, 2006)

	Male			Female		

	OR	*p*	[95% CI]	OR	*p*	[95% CI]
Age						
50–54	1			1		
55–59	0.76	0.734	(0.16–3.60)	3.12	0.011	(1.29–7.52)
60–64	0.64	0.592	(0.12–3.29)	2.25	0.074	(0.92–5.48)
65–69	0.95	0.948	(0.21–4.39)	1.87	0.164	(0.77–4.5)
70–74	0.68	0.662	(0.12–3.88)	1.57	0.359	(0.6–4.16)
75–79	0.35	0.231	(0.06–1.94)	5.19	0.001	(1.9–14.14)
80 plus	0.92	0.923	(0.15–5.52)	5.04	0.004	(1.7–14.93)
Waist/hip ratio						
Normal				1		
Substantially increased				2.68	0.004	(1.37–5.26)
Education level						
No education				1		
Less than 6 years				2.31	0.025	(1.11–4.82)
Six years or more				1.07	0.873	(0.46–2.49)
Waist circumference						
Normal	1					
Increased	4.28	0.018	(1.29–14.22)			
Substantially increased	3.71	0.022	(1.21–11.41)			
Alcohol consumption						
Never drink	1					
Past drink	1.39	0.569	(0.45–4.29)			
Currently drink	5.21	0.009	(1.50–18.13)			
Household asset score						
Lowest	1					
Middle low	1.56	0.576	(0.33–7.4)			
Middle	2.22	0.317	(0.47–10.57)			
Middle high	4.13	0.061	(0.94–18.23)			
Highest	1.39	0.657	(0.33–5.92)			

The 191 participants who had used a health facility in the last year accounted between them for 448 visits. Many had used health facilities only once, but 25 participants (13.0% of 191) were responsible for 45.9% of all reported visits. Table 5 shows the risk factors of hypertension separately for men and women. In men, a larger waist circumference (increased waist circumference odds ratio [OR] 4.3; 95% CI 1.3–14.2, substantially increased waist circumference OR 3.7; 95% CI 1.2–11.4) and currently drinking alcohol (OR 5.2; 95% CI 1.5–18.1) were associated with a higher risk of hypertension. In women, age had a U-shaped association with hypertension (OR aged 55–59 years 3.1, 95% CI 1.3–7.5; OR aged 70–74 years 1.6, 95% CI 0.6–4.2; and OR aged 80 years plus 5.0, 95% CI 1.7–14.9). Other factors associated with hypertension in women were a higher waist–hip ratio (OR 2.7; 95% CI 1.4–5.2) and level of education where those with less than 6 years of formal education were at higher risk than those without any formal education (OR 2.3; 95% CI 1.1–4.8).

## Discussion

In general, this group of older people reports a good quality of life and good or moderately good health. Nevertheless, there was a high prevalence of self-reported health conditions, most notably hypertension (31%) and musculoskeletal pain (42%). Although nearly a third of participants reported being previously diagnosed with a non-communicable disease, around two fifths (43%) reported that they had not needed any health care in the last 12 months.

An important limitation of this study is that apart from blood pressure, we have not been able to confirm the self-reported diagnoses of the non-communicable diseases. The results for hypertension suggest that this self-report may represent an underestimation of the true situation. On the other hand, the self reported approach to measure smoking and drinking may lack specifity and therefore could tend to overestimate levels of risk. Another limitation is the relatively small number of participants, which limits the potential for exploring the relationship between some of the variables.

The individuals in this study were born during the Apartheid era and were almost certainly disadvantaged in the early years of their lives, with low survival. By age 50, the youngest age for inclusion in this study, those who survive and were included in the study may represent a selectively healthier group. While we are confident that this random sample represents the older population of the Agincourt sub-district, the sub-district itself may not represent the rural population of South Africa. The Ehlanzeni district, where Agincourt is situated, is the poorest district in Mpumalanga Province, as assessed by the proportion of individuals living in households where the income is below the monthly income defined as that needed to sustain a household. In Ehlanzeni district, 54.5% of individuals were living in such households, compared with the national rate of 40.7% (28).

The use of anti-retrovirals is unlikely to have had any effect on the health of the individuals in this study. In 2006, when this study was conducted, very few people knew their HIV status and even fewer were using anti-retroviral treatment (ART). These drugs were only available from late 2005 and only in two hospitals between 25 and 60 km from the Agincourt sub-district.

A key finding is the low level of health care use by older people, despite the prevalence of non-communicable diseases and HIV in this population. Previous research has also found low levels of health care use in South Africa (12, 29, 30). Forty-three percent of participants reported that they had not needed care in the last year. In comparison, a study in Botswana found that 42% of the elderly had used health care facilities during the month previous to the interview (31). It is likely that there is considerable unmet and unrecognised need for health care. This is supported by the high proportion of individuals in the study who were unaware they had hypertension. Adherence may also be a problem. A study in Tanzania found that even when older people are referred to a health facility after screening for hypertension, a low percentage of them used Western health care. However, hypertensive treatment was not free, as it is in South Africa (32).

While the figures on use of medication in those reporting diabetes and other major conditions might indicate good adherence to medication, they should be interpreted with considerable caution since they only indicate some medication use within 12 months. We have no information on duration of use or on whether the medication used was prescribed by a health professional. It is routine in clinics in this area to provide no more than one month's supply of medication at a time, and this may be a barrier to long term adherence.

As part of improving treatment for this group of people, a better drug supply in the primary health care facilities would be necessary to allow nurses to provide treatment for more than 1 month, which is still the routine in most clinics in this rural area.

The significantly higher level of respiratory disease reported by men may relate to smoking habits or prolonged stay on the mines and underground, a long standing feature of the South African and regional economy.

When we measured the blood pressure of participants, we found a very high prevalence of hypertension (57%), much higher than self-reported. One earlier national study carried out in 1998 in South Africa reported a combined rural and urban prevalence of 21% in the population aged 15 plus, with 44% in African men aged 45 years and older, and 50% in African women of the same age (12). As in our results, the earlier study found that the high levels of hypertension came with low levels of awareness of the condition (10).

Our study shows high levels of hypertension in a rural South African setting, even higher than those found 8 years ago in urban areas. These findings support earlier predictions that hypertension levels were going to increase in the early 2000s and indicate that the problem of hypertension in older people is not just an urban phenomenon but is universal (3).

We found that women were more likely than men to be aware of their blood pressure status, which may well be explained by their more frequent use of health services. This corroborates findings in other African settings where women had higher levels of awareness, treatment, and control of hypertension (2, 3).

Gender differences related to health and health-seeking behaviour are well characterised in this study. In a previous study (33), women reported higher levels of disability and poorer quality of life. Our study corroborates other reports that women use health facilities more than men and are more aware of their health situation (10).

Women were very much more likely than men to be obese. A similar gendered pattern of body size has been reported previously in southern Africa (34–36), but no credible explanation has yet been put forward. Despite these findings, there was no difference between men and women either in the prevalence of hypertension or in mean levels of blood pressure. A recent systematic review of hypertension in sub-Saharan Africa found similar findings to those reported here. There were low levels of detection, treatment, and control and only minimal differences between men and women in the prevalence of hypertension (3).

As emphasised by the South Africa Minister of Health, Aaron Motsoaledi, in his talk to the Board of Health Care Funders in 2011, South Africa is running ‘a health care system that is not working’, and, he argued, ‘the solution lies in re-engineering primary health care’ (37). It remains to be seen whether a new system of integrated chronic care will successfully meet the currently unmet needs of a growing population of older people with chronic disease. The Department of Health is piloting an integrated chronic care system in primary health care clinics in Bushbuckridge and the evaluation of this pilot will provide important information to guide the development of the South African primary health care system.

### Ethical clearance

Ethical clearance for the MRC/WITS Rural Public Health and Health Transitions Research Unit's (Agincourt) Health and socio-Demographic Surveillance System was granted by the Committee for Research on Human Subjects (Medical) University of the Witwatersrand, Johannesburg, South Africa (Ref M960720).

Ethical clearance for the Agincourt-INDEPTH Study on Global Ageing and Adult Health was given by the Committee for Research on Human Subjects (Medical) University of the Witwatersrand, Johannesburg, South Africa (Ref R14/49).

## References

[1] Nissinen A, Bothig S, Granroth H, Lopez AD (1988). Hypertension in developing countries. World Health Stat Q.

[2] Fuentes R, Ilmaniemi N, Laurikainen E, Tuomilehto J, Nissinen A (2000). Hypertension in developing economies: a review of population-based studies carried out from 1980 to 1998. J Hypertens.

[3] Addo J, Smeeth L, Leon DA (2007). Hypertension in sub-saharan Africa: a systematic review. Hypertension.

[4] Norman R, Gaziano T, Laubscher R, Steyn K, Bradshaw D (2007). Estimating the burden of disease attributable to high blood pressure in South Africa in 2000. S Afr Med J.

[5] Mayosi BM, Flisher AJ, Lalloo UG, Sitas F, Tollman SM, Bradshaw D (2009). The burden of non-communicable diseases in South Africa. Lancet.

[6] United Nations Political declaration of the High-level Meeting of the General Assembly on the Prevention and Control of the Non-communicable diseases.

[7] (2011). South African declaration on the Prevention and Control of Non-communicable diseases. http://www.health.uct.ac.za/usr/health/research/groupings/cdia/downloads/SA_NCD_Declaration.pdf.

[8] Tollman SM, Kahn K, Sartorius B, Collinson MA, Clark SJ, Garenne ML (2008). Implications of mortality transition for primary health care in rural South Africa: a population-based surveillance study. Lancet.

[9] Statistics South Africa (2011). Mid year population estimates. http://www.statssa.gov.za/publications/P0302/P03022011.pdf.

[10] Steyn K, Gaziano TA, Bradshaw D, Laubscher R, Fourie J (2001). Hypertension in South African adults: results from the demographic and health survey, 1998. J Hypertens.

[11] Steyn K, Bradshaw D, Norman R, Laubscher R (2008). Determinants and treatment of hypertension in South Africans: the first demographic and health survey. S Afr Med J.

[12] Levitt NS, Steyn K, Dave J, Bradshaw D (2011). Chronic noncommunicable diseases and HIV-AIDS on a collision course: relevance for health care delivery, particularly in low-resource settings – insights from South Africa. Am J Clin Nutr.

[13] Gómez-Olivé FX, Day CBP, Massyn N, Padarath A, English R (2012). Chronic care: hypertension and diabetes mellitus detection rate indicators. District health barometer 2010/2011.

[14] Coovadia H, Jewkes R, Barron P, Sanders D, McIntyre D (2009). The health and health system of South Africa: historical roots of current public health challenges. Lancet.

[15] Moshabela M, Pronyk P, Williams N, Schneider H, Lurie M (2011). Patterns and implications of medical pluralism among HIV/AIDS patients in rural South Africa. AIDS Behav.

[16] Republic of South Africa (2011). Policy on National Health Insurance.

[17] Kahn K, Tollman SM, Collinson MA, Clark SJ, Twine R, Clark BD (2007). Research into health, population and social transitions in rural South Africa: data and methods of the Agincourt Health and Demographic Surveillance System. Scand J Public Health Suppl.

[18] Collinson MA, Mokoena O, Mgiba N, Kahn K, Tollman SM, Garenne M, Sankoh O, Kahn K, Mwageni E (2002). Agincourt Demographic Surveillance System (Agincourt DSS). Population and health in developing countries.

[19] Hundt GL, Stuttaford M, Ngoma B (2004). The social diagnostics of stroke-like symptoms: healers, doctors and prophets in Agincourt, Limpopo Province, South Africa. J Biosoc Sci.

[20] World Health Organization Health Statistics and Health Information Systems, questionnaires. CSda.

[21] Collinson MA, Clark SJ, Gerritsen AM, Byass P, Kahn K, Tollman SM (2009). The dynamics of poverty and migration in a rural South African community, 2001–2005. http://www.csss.washington.edu/Papers/wp92.pdf.

[22] Hargreaves JR, Collinson MA, Kahn K, Clark SJ, Tollman SM (2004). Childhood mortality among former Mozambican refugees and their hosts in rural South Africa. Int J Epidemiol.

[23] (1993). Study protocol for the World Health Organization project to develop a Quality of Life assessment instrument (WHOQOL). Qual Life Res.

[24] (1995). The World Health Organization Quality of Life assessment (WHOQOL): position paper from the World Health Organization. Soc Sci Med.

[25] (1998). The World Health Organization Quality of Life Assessment (WHOQOL): development and general psychometric properties. Soc Sci Med.

[26] Ustun TB, Chatterji S, Kostanjsek N, Rehm J, Kennedy C, Epping-Jordan J (2010). Developing the World Health Organization Disability Assessment Schedule 2.0. Bull World Health Organ.

[27] Chobanian AV, Bakris GL, Black HR, Cushman WC, Green LA, Izzo JL (2003). The Seventh Report of the Joint National Committee on Prevention, Detection, Evaluation, and Treatment of High Blood Pressure: The JNC 7 Report. JAMA.

[28] (2009). Mpumalanga Economic Profile, Department of Economic Development, Environment and Tourism. Mpumalanga Provincial Government.

[29] Connor MD, Thorogood M, Casserly B, Dobson C, Warlow CP (2004). Prevalence of stroke survivors in rural South Africa: results from the Southern Africa Stroke Prevention Initiative (SASPI) Agincourt field site. Stroke.

[30] Nyirenda M, Chatterji S, Falkingham J, Mutevedzi P, Hosegood V, Evandrou M (2012). An investigation of factors associated with the health and well-being of HIV-infected or HIV-affected older people in rural South Africa. BMC Publ Health.

[31] Clausen F, Sandberg E, Ingstad B, Hjortdahl P (2000). Morbidity and health care utilisation among elderly people in Mmankgodi village, Botswana. J Epidemiol Community.

[32] Bovet P, Gervasoni JP, Mkamba M, Balampama M, Lengeler C, Paccaud F (2008). Low utilization of health care services following screening for hypertension in Dar es Salaam (Tanzania): a prospective population-based study. BMC Publ Health.

[33] Gomez-Olive FX, Thorogood M, Clark BD, Kahn K, Tollman SM (2010). Assessing health and well-being among older people in rural South Africa. Glob Health Action.

[34] Bourne LT, Lambert EV, Steyn K (2002). Where does the black population of South Africa stand on the nutrition transition?. Publ Health Nutr.

[35] Reddy SP, Resnicow K, James S, Funani IN, Kambaran NS, Omardien RG (2012). Rapid increases in overweight and obesity among South African adolescents: comparison of data from the South African National Youth Risk Behaviour Survey in 2002 and 2008. Am J Publ Health.

[36] Dalal S, Beunza JJ, Volmink J, Adebamowo C, Bajunirwe F, Njelekela M (2011). Non-communicable diseases in sub-Saharan Africa: what we know now. Int J Epidemiol.

[37] Health minister moots ‘three levels of care’. Health System Trust Bulletin [Internet].

