# Corrigendum

**DOI:** 10.3402/gha.v7.24080

**Published:** 2014-03-13

Regarding the paper titled ‘Self-reported health and health care use in an ageing population in the Agincourt sub-district of rural South Africa’ by *Francesc Xavier Gómez-Olivé, Margaret Thorogood, Benjamin Clark, Kathleen Kahn, Stephen Tollman*

Published in *Global Health Action* (Supplement 1, 2013) 24 Jan 2013. Citation: Glob Health Action 2013, **6**: 19305 - http://dx.doi.org/10.3402/gha.v6i0.19305

The last sentence of the third paragraph in the Results section is incorrect:

It currently reads: “Of the 262 women who had measured hypertension, 81 (30.9%) reported that they had hypertension, while of the 116 men with measured hypertension only 22 (19%) reported that they had hypertension (p=0.025)”

This sentence should read: “*Of the 149 women who had measured hypertension, 81 (54.4%) reported that they had hypertension, while of the 66 men with measured hypertension only 22 (33.3%) reported that they had hypertension (p=0.025)*”

The complete paragraph is displayed below

We compared self-reported hypertension with measured blood pressure. Of the 255 participants who did not report that they had hypertension, high blood pressure levels compatible with hypertension were present in 112 (43.9%) (p<0.001). Half of the 20 participants who reported hypertension but were normotensive when their blood pressure was measured were on treatment for hypertension. The positive predictive value of a self-reported diagnosis of hypertension was 91.9%, but the negative predictive value of a self-reported normal blood pressure was only 43.9%. Awareness of diagnosis of hypertension varied with gender. Of the 149 women who had measured hypertension, 81 (54.4%) reported that they had hypertension, while of the 66 men with measured hypertension only 22 (33.3%) reported that they had hypertension (p=0.025).

